# Quantitative Susceptibility Mapping-Derived Radiomic Features in Discriminating Multiple Sclerosis From Neuromyelitis Optica Spectrum Disorder

**DOI:** 10.3389/fnins.2021.765634

**Published:** 2021-12-03

**Authors:** Zichun Yan, Huan Liu, Xiaoya Chen, Qiao Zheng, Chun Zeng, Yineng Zheng, Shuang Ding, Yuling Peng, Yongmei Li

**Affiliations:** ^1^Department of Radiology, The First Affiliated Hospital of Chongqing Medical University, Chongqing, China; ^2^GE Healthcare, Shanghai, China

**Keywords:** multiple sclerosis, neuromyelitis optica spectrum disorder, quantitative susceptibility mapping, radiomics, discrimination

## Abstract

**Objectives:** To implement a machine learning model using radiomic features extracted from quantitative susceptibility mapping (QSM) in discriminating multiple sclerosis (MS) from neuromyelitis optica spectrum disorder (NMOSD).

**Materials and Methods:** Forty-seven patients with MS (mean age = 40.00 ± 13.72 years) and 36 patients with NMOSD (mean age = 42.14 ± 12.34 years) who underwent enhanced gradient-echo T_2_*-weighted angiography (ESWAN) sequence in 3.0-T MRI were included between April 2017 and October 2019. QSM images were reconstructed from ESWAN, and QSM-derived radiomic features were obtained from seven regions of interest (ROIs), including bilateral putamen, globus pallidus, head of the caudate nucleus, thalamus, substantia nigra, red nucleus, and dentate nucleus. A machine learning model (logistic regression) was applied to classify MS and NMOSD, which combined radiomic signatures and demographic information to assess the classification accuracy using the area under the receiver operating characteristic (ROC) curve (AUC).

**Results:** The radiomics-only models showed better discrimination performance in almost all deep gray matter (DGM) regions than the demographic information-only model, with the highest AUC in DN of 0.902 (95% CI: 0.840–0.955). Moreover, the hybrid model combining radiomic signatures and demographic information showed the highest discrimination performance which achieved the AUC of 0.927 (95% CI: 0.871–0.984) with fivefold cross-validation.

**Conclusion:** The hybrid model based on QSM and powered with machine learning has the potential to discriminate MS from NMOSD.

## Introduction

Multiple sclerosis (MS) and neuromyelitis optica spectrum disorder (NMOSD) are two major inflammatory demyelinating diseases of the central nervous system (Noseworthy et al., 2000; Wingerchuk et al., 2007). The discovery of the aquaporin-4 antibody (AQP4-Ab) indicates that NMOSD is an inflammatory demyelinating disease independent of MS (Sinnecker et al., 2012; Jurynczyk et al., 2015; Liu et al., 2015; Wingerchuk et al., 2015). NMOSD shows a high prevalence for Asian people. MS and NMOSD share similar clinical manifestations and image features because they both have damages in the optic nerve, spinal cord, and brain, making it difficult to distinguish each other in clinic practice (Huh et al., 2014). Although AQP4-Ab is a common contributor for NMOSD, some NMOSD patients may show a negative autoantibody in cerebrospinal fluid and serum specimens, resulting in incorrect clinical diagnosis and mistreatment (Lennon et al., 2004; Calabrese et al., 2012; Jarius and Wildemann, 2013). In particular, NMOSD sometimes is more serious than MS, and the recommended treatment for MS could exacerbate NMOSD progression (Palace et al., 2010; Jarius and Wildemann, 2013). Therefore, it is vital to diagnose between these two diseases accurately.

Magnetic resonance imaging (MRI) is considered a highly sensitive imaging modality in identifying MS and NMOSD patients. However, conventional MRI scans could not meet the need for higher efficiency of differential diagnosis due to atypical lesion distribution in MS and NMOSD. Therefore, advanced neuroimaging techniques have been used to find more disease characterization and improve optimal therapies. Iron is primarily found in the deep gray matter (DGM) structures within the brain, including globus pallidus (GP), putamen (PUT), head of caudate nucleus (HCN), red nucleus (RN), substantia nigra (SN), thalamic (THA), and the dentate nucleus (DN) (Haacke et al., 2005; Chen et al., 2012). Particularly, previous studies have demonstrated that quantitative susceptibility mapping (QSM), as a novel image sequence, can help to distinguish MS or NMOSD patients from healthy individuals due to the abnormal patterns of iron deposition (Langkammer et al., 2013; Cobzas et al., 2015; Doring et al., 2016; Hagemeier et al., 2017; Zivadinov et al., 2018), which indicated that iron deposition was important in MS and NMOSD pathology. The different QSM-derived magnetic susceptibility values in the DGM between MS and NMOSD were reported by Pudlac et al. (2020). Therefore, QSM may serve as a useful imaging biomarker in guiding early identification and differentiation between MS and NMOSD. However, the mere magnetic susceptibility of the tissue does not ideally account for its iron content because iron occurs in forms of different magnetic properties, and related studies are still limited.

Recently, radiomics extracts the abundant high-throughput radiomic features from conventional medical images and has been recognized as an emerging and attractive research technology, which has shown remarkable success in characterizing tumor phenotypes (Yip and Aerts, 2016). Using hand-crafted features in the regions of interest (ROIs), radiomics makes it possible to extract high-dimensional and mineable features, which can be used for pathophysiology classification in clinical practice (Lambin et al., 2012; Gillies et al., 2016; Yip and Aerts, 2016). Therefore, the QSM sequence was applied to extract radiomic features from DGM regions. Then, a discrimination machine learning model that incorporates the QSM-derived radiomic features and demographic information was performed to classify between MS and NMOSD.

## Materials and Methods

### Standard Protocol Approvals, Registrations, and Patient Consents

This retrospective study has been approved by the Institutional Review Board of the First Affiliated Hospital of Chongqing Medical University, Chongqing, China, and written informed consent was obtained from each participant before MRI scans.

### Participants

**FIGURE 1:**
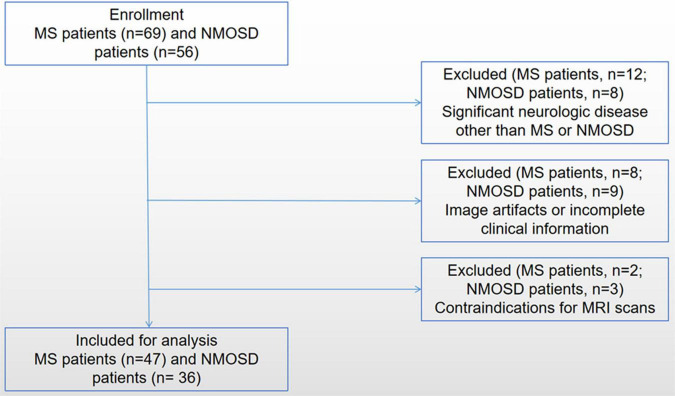
The flowchart shows the enrollment of MS and NMOSD patients.

Patients diagnosed with MS or NMOSD were enrolled from the Department of Radiology, the First Affiliated Hospital of Chongqing Medical University, between April 2017 and October 2019 (Figure 1). Patients who met the following inclusion criteria were chosen: (1) patients have received a confirmed diagnosis of NMOSD based on the standard diagnosis criteria (Wingerchuk et al., 2015) and confirmed MS according to the 2017 McDonald criteria (Thompson et al., 2018); (2) patients have undergone enhanced gradient-echo T_2_*-weighted angiography (ESWAN) sequence imaging on the same 3.0-T scanner with standardized study protocol; and (3) patients have been in remission (relapse-free for at least 4 weeks) and have no treatment of disease-modified medications within 4 weeks before MRI scans. The exclusion criteria were (1) significant neurologic disease other than MS or NMOSD; (2) image artifacts or incomplete clinical information; and (3) contraindications for MRI scans.

Demographic and clinical data including patient age, sex, disease duration, and Expanded Disability Status Scale (EDSS) score were recorded.

### Magnetic Resonance Imaging Acquisitions

All patients underwent an MRI scan of the brain on a 3.0-T system (GE Medical Systems, Milwaukee, WI, United States) using an eight-channel phased-array head coil. The standard protocol for MS studies was performed including a conventional axial 2D dual-echo proton density (PD)-T_2_-weighted imaging (T_2_WI) [repetition time (TR) = 2,900 ms, echo time (TE)1 = 25 ms, TE2 = 93 ms, echo train length [ETL) = 12, matrix size = 256 × 192] and 2D fluid-attenuated inversion recovery (FLAIR) (TR = 2,050 ms, TE = 24 ms, TI = 750 ms, matrix size = 256 × 256). All axial scans were taken from the roof of the skull to the foramen magnum [slice thickness = 5 mm, slice skip = 0 mm, field of view (FOV) = 24 cm × 24 cm].

ESWAN data were acquired with eight echoes using the following parameters: TR = 60 ms, TE = 6 ms, number of excitation (NEX) = 0.75, FOV = 22 cm × 22 cm, matrix size = 448 × 320, receiver bandwidth = + 62.5 kHz, and flip angle = 20°. The sequence was acquired with 2-mm-thick contiguous sections and no space. All scans were oriented parallel to the anterior–posterior commissural (AC-PC) line with 56–64 locations on the middle sagittal plane and covered the entire brain area.

### Quantitative Susceptibility Mapping Reconstructions

QSM reconstructions were performed using a MATLAB R2013a-based susceptibility imaging software (STISuite^1^). The corrected and combined phase images were acquired by weighting the magnitude of the corresponding channel with the vendor-provided combination method and were unwrapped using a Laplacian-phase method. Then, phase-unwrapped images were used to remove the background field using the V-SHARP method (Schweser et al., 2011). In order to reduce extreme streaking artifacts caused by large veins, susceptibility maps were generated in the process of field-to-susceptibility inversion by using an improved sparse linear equation and least-square algorithm (streaking artifact reduction for QSM, STAR-QSM).

### Quantitative Susceptibility Mapping Atlas Registration and Regions of Interest Segmentation

**FIGURE 2:**
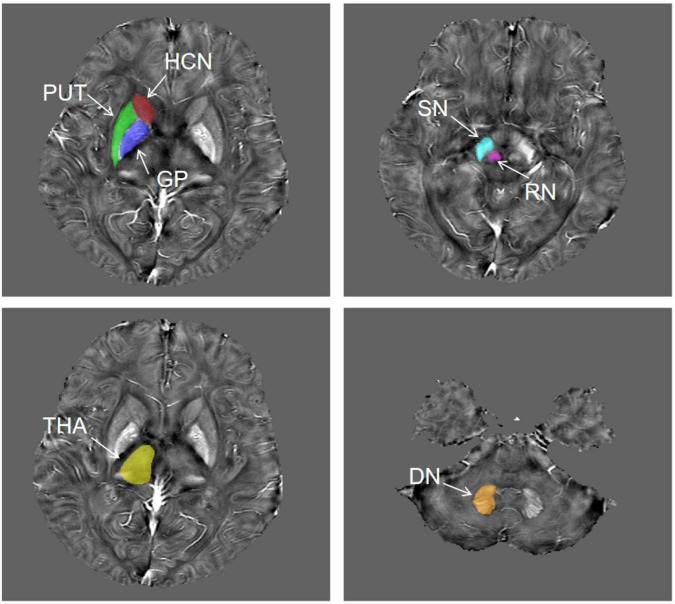
The representative unilateral outlines of the defined ROIs. The ROIs included the putamen (PUT), globus pallidus (GP), head of the caudate nucleus (HCN), thalamus (THA), substantia nigra (SN), red nucleus (RN), and dentate nucleus (DN) bilaterally.

The QSM atlas is available in the STISuite V3.0 software package and can be downloaded at, (see text footnote 1) (Zhang et al., 2018). The original QSM data for each participant were registered to the QSM atlas by using the FMRIB Software Library (FSL^2^). Furthermore, seven ROIs for each patient, including bilateral PUT, GP, HCN, THA, SN, RN, and DN, were segmented on QSM images by using Statistical Parametric Mapping (SPM) software based on MATLAB R2013a. Figure 2 shows the representative unilateral outlines of the defined ROIs.

### Feature Extraction and Selection

**FIGURE 3:**
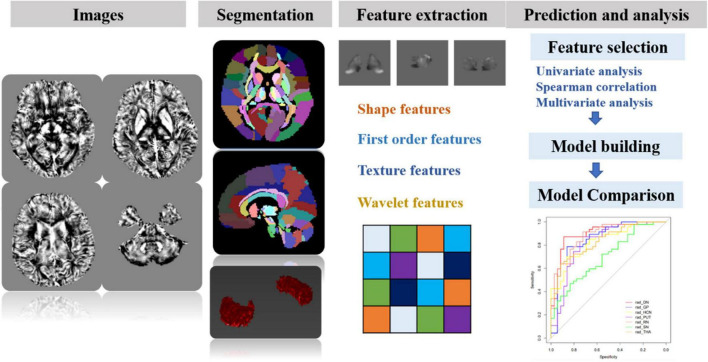
The schematic flowchart of processing steps. Step 1: QSM reconstructions were performed. Step 2: after QSM atlas registration, seven ROIs were segmented on QSM images. Step 3: four sets of most representative features were chosen for generalizing and optimizing the model. Step 4: after feature selection, the prediction model for differentiating MS and NMOSD was built by combining radiomic features and clinical information. Furthermore, the classification performance was assessed with AUC using fivefold cross-validation.

Before the feature extraction, an isotropic voxel was resampled into 1 mm × 1 mm × 1 mm with linear interpolation for the purpose of normalizing the geometry of MR images. A total of 874 radiomic features were extracted by using the in-house software AK (Artificial Intelligence Kit, version 3.3.0, GE Healthcare, Chicago, IL, United States), including four different sets of features: (1) shape features, (2) first-order features, (3) textural features, and (4) transform features including wavelet and Laplace of Gaussian. The shape-based features were measured using the shape descriptors of three-dimensional size and shape of the ROI. First-order statistical features were applied to describe the distribution of voxel intensities. Texture features used a matrix to represent the spatial heterogeneity of the intensity level. To further investigate the intra-ROI heterogeneity, wavelet filters and Laplace transformation were applied to the original images to convert original images to versions focused on information at different scales. Most of the extracted features obey the imaging biomarker standardization initiative (Zwanenburg et al., 2020). Before the feature selection, the abnormal or missing data were replaced by the median, and the standardization was performed to eliminate dimension differences. Firstly, the univariate analysis was applied to choose the significant features with a *p-*value less than 0.05. Next, the *spearman* correlation analysis between features was used with the cutoff of 0.9, reducing the features redundancy. Then, the multivariate logistic analysis with the Akaike information criterion was used to select the most representative features. Finally, the multivariable logistic regression model with fivefold cross-validation was established. The flowchart of the processing steps is shown in Figure 3.

### Model Establishment and Performance Evaluation

The prediction models for differentiating MS and NMOSD were built with the logistic regression by the radiomic features only, demographic information (patient age, sex, disease duration, and EDSS score) and combining both above, respectively.

The area under the curves (AUC), sensitivity, specificity, and accuracy were used to evaluate the classification performance of the models. The classification performance was assessed with AUC using fivefold cross-validation. The DeLong test was used to compare the different radiomics models.

### Statistical Analysis

Statistical analysis was performed by using the in-house software IPMs (IPM Statistics, version 2.4.0, GE Healthcare). Discrete data encoding the sexes of the patients were analyzed using *Pearson’s chi-square* test. Two-sided two-sample *t-*tests or *Mann–Whitney U* tests were used to assess between-group differences for continuous demographic or clinical data, depending on whether they were normally distributed using the *Lilliefors* test. A two-tailed *p*-value < 0.05 represents the significant difference.

## Results

### Demographic Data Analysis

The demographic and clinical characteristics of the enrolled patients are summarized in Table 1. Demographic information was collected at the time of visit including patient age, sex, disease duration, and EDSS score.

**TABLE 1:** The demographic and clinical data of participants.

Characteristics	MS	NMOSD	*p-*value
No. of patients	47	36	
Sex (male/female)	18/29	6/30	0.031^a^
Age (years)	40.00 ± 13.72	42.14 ± 12.34	0.464^b^
Disease duration (years)	5.42 (2.07, 9.19)	3.88 (1.29, 9.06)	0.301^c^
EDSS	2.00 (1.00, 3.00)	2.50 (2.00, 4.78)	0.002^c^

*EDSS, Expanded Disability Status Scale; MS, multiple sclerosis; NMOSD, neuromyelitis optica spectrum disorder.*

*^a^p-values obtained using the Pearson’s chi-square test.*

*^b^p-values obtained using two-sample two-tailed t-tests.*

*^c^p-values obtained using two-tailed Mann–Whitney U-test.*

The NMOSD patients showed a greater female predominance, and higher EDSS scores, than the MS patients.

### Radiomic Feature-Only Selection and Model Building

In this study, 874 radiomic features were extracted from the ROIs. First, with univariate analysis, the seven DGM regions (HCN, DN, GP, PUT, RN, SN, and THA) remained 51, 59, 206, 49, 78, 9, and 192 features, respectively. Next, the number of the 19, 21, 57, 27, 33, 7, and 67 features were then selected with the *Spearman* correlation analysis. Finally, with the multivariate analysis, the radiomic features were reduced to several most predictive features (HCN: 4, DN: 4, GP: 3, PUT: 3, RN: 5, SN: 2, THA: 3) in each region. Moreover, the selected radiomic features in different regions are shown in Table 2. The machine learning models with logistic regression were established with fivefold cross-validation.

**TABLE 2:** The selected radiomic features with different regions.

ROIs	Features
DN	original_glrlm_GrayLevelNonUniformity wavelet–LHH_glszm_GrayLevelVariance wavelet–HLL_glszm_GrayLevelVariance log–sigma-1–0-mm–3D_glszm_GrayLevelNonUniformity
GP	wavelet–LLH_glrlm_ShortRunHighGrayLevelEmphasis wavelet–LHL_gldm_DependenceEntropy wavelet–HLL_glszm_ZoneVariance log–sigma–1–0–mm–3D_glszm_ZoneEntropy
HCN	wavelet–HLH_glcm_JointAverage wavelet–HHL_glszm_HighGrayLevelZoneEmphasis wavelet–LLH_gldm_SmallDependenceHighGrayLevelEmphasis
PUT	wavelet–HHL_glrlm_ShortRunHighGrayLevelEmphasis wavelet–HHL_glcm_Imc1 wavelet–LLH_glszm_SizeZoneNonUniformityNormalized
RN	wavelet–LHH_gldm_SmallDependenceHighGrayLevelEmphasis wavelet–HLL_firstorder_Kurtosis wavelet–HLL_gldm_SmallDependenceHighGrayLevelEmphasis wavelet–HHL_glrlm_HighGrayLevelRunEmphasis wavelet–HLL_glszm_GrayLevelNonUniformity
SN	wavelet–LLH_glrlm_ShortRunHighGrayLevelEmphasis wavelet–HLL_firstorder_Skewness
THA	wavelet–LHL_glrlm_HighGrayLevelRunEmphasis wavelet–LLL_glrlm_ShortRunHighGrayLevelEmphasis wavelet–HHL_firstorder_Range

*DN, dentate nucleus; GP, globus pallidus; HCN, head of the caudate nucleus; PUT, putamen; RN, red nucleus; SN, substantia nigra; THA, thalamus.*

### Model Evaluation

**FIGURE 4:**
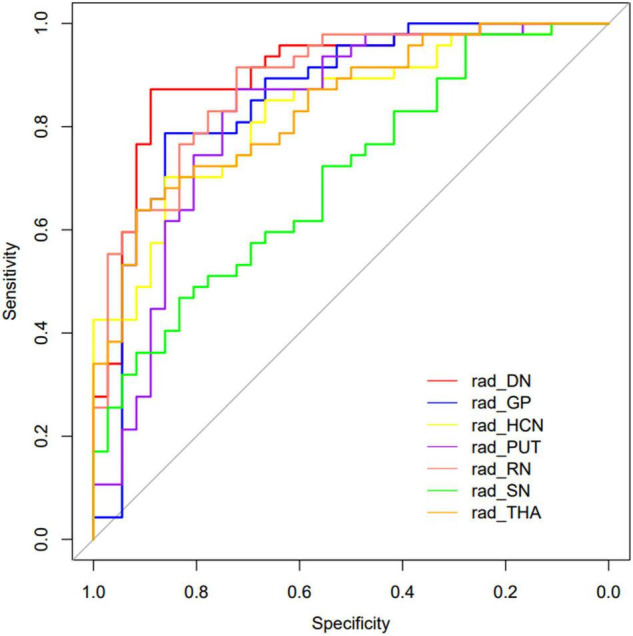
The curves of the different model were shown. Different colors represented the model of different DGM regions.

The performance of the prediction models assessed with the AUC, sensitivity, specificity, and accuracy were calculated. The DGM regions except for SN (AUC: 0.702, 95% CI: 0.603–0.793) had a better discrimination performance than the demographic data-only model and were more than 0.80. The model comparisons with the DeLong test were performed, as shown in Table 3; the results showed a significant difference that the *p*-value was 0.002 between DN and SN, 0.016 between GP and SN, and 0.004 between RN and SN, and there were no significant differences between other models. The DN reflected the best performance in all DGM regions with the AUC of 0.902 (95% CI: 0.840–0.955), a sensitivity of 0.851, a specificity of 0.889, and an accuracy of 0.867. All radiomics-only models’ discrimination performance including AUC, sensitivity, specificity, and accuracy are shown in Table 4. The receiver operating characteristic curve (ROC) of all DGM regions is shown in Figure 4.

**TABLE 3:** The DeLong test for the model comparisons.

Model	DeLong test					
	GP	HCN	PUT	RN	SN	THA
DN	0.434	0.187	0.216	0.754	0.0018*	0.289
GP		0.662	0.599	0.596	0.016*	0.727
HCN			0.877	0.312	0.083	0.891
PUT				0.289	0.107	0.743
RN					0.0042*	0.406
SN						0.042

*Represents significant comparison differences between models.

**TABLE 4:** Comparison of the different radiomics models.

Model	AUC (95% CI)	Sensitivity	Specificity	Accuracy
DN	0.902 (0.84, 0.955)	0.851	0.889	0.867
GP	0.856 (0.773, 0.928)	0.766	0.861	0.807
HCN	0.830 (0.759, 0.899)	0.681	0.861	0.759
PUT	0.821 (0.737, 0.901)	0.851	0.722	0.795
RN	0.885 (0.823, 0.941)	0.894	0.722	0.819
SN	0.702 (0.603, 0.793)	0.447	0.833	0.614
THA	0.838 (0.763, 0.906)	0.617	0.917	0.747

*AUC, area under the receiver operating characteristic curve.*

**FIGURE 5:**
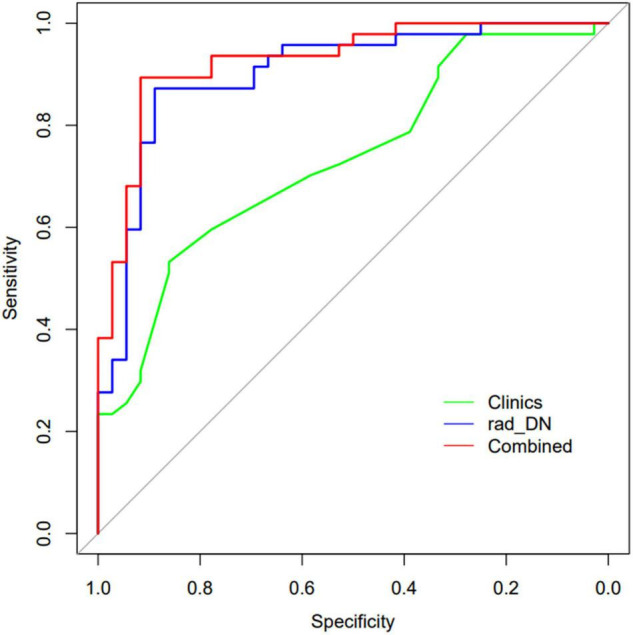
The ROCs of the demographic information-only model, radiomics-only model of DN, and the combined model are shown.

The demographic information-only model was built and showed an AUC of 0.733 (95% CI: 0.639–0.818), a sensitivity of 0.511, a specificity of 0.861, and an accuracy of 0.663. We used QSM-derived radiomic features, plus four demographic variables: sex, age, disease duration, and EDSS score to build a regression model to differentiate MS from NMOSD. The combined model showed a greater discrimination performance than the models mentioned above with an AUC of 0.927 (95% CI: 0.871–0.984). The ROC of the demographic information-only model, radiomics-only model of DN, and combined model are shown in Figure 5.

## Discussion

In the present study, we investigated the great performances of QSM-derived radiomic features in discriminating MS from NMOSD. Combining the radiomic features and demographic information to establish the hybrid model, it showed a higher discrimination performance, which can help the clinical differential diagnosis of these two diseases.

The diagnostic criteria of MS and NMOSD are constantly updated and recognized by the public, but MS showed inflammation, axonal loss, oligodendrocyte damage, gliosis, macrophage infiltration, microglia, and neurodegeneration, while NMOSD presented a perivascular immunoglobulin deposition and complement activation. Thus, it is still challenging to differentiate and diagnose these two diseases in clinical practice. Radiomics methods can provide us with more information. In previous studies, radiomics methods were based on traditional MRI techniques to differentiate MS from NMOSD. Liu et al. (2019) extracted radiomic features from the spinal cord lesions on sagittal T_2_WI, and Ma et al. (2019) extracted the radiomics from lesions on T_2_WI to differentiate MS from NMOSD. There are different levels of iron in the brain. Therefore, the traditional MRI scans are based on the demyelination and inflammation of the lesions, but the iron deposition is also the pathological change of MS and NMOSD. Therefore, iron deposition is also essential in the differential diagnosis of these two diseases. Iron is an essential trace element for the human body which keeps many enzymes active, such as neurotransmitter synthesis, myelin formation, and mitochondrial energy metabolism. Its homeostasis is crucial for the normal physiological function of the brain. However, excess free iron catalyzes the generation of a large number of free radicals, which enhances oxygen toxicity and leads to oxidative hyperactivity, thus participating in the pathogenesis of MS and NMOSD (Doring et al., 2016; Carocci et al., 2018; Zivadinov et al., 2018). As an advanced MRI technique for quantitative iron deposition, QSM-related studies (Chen et al., 2012; Langkammer et al., 2013; Rudko et al., 2014; Cobzas et al., 2015; Doring et al., 2016; Elkady et al., 2017; Hagemeier et al., 2017; Zivadinov et al., 2018; Pudlac et al., 2020) have also studied and confirmed the differences between the two diseases. Therefore, the differences in iron deposition are highly likely to change the image characteristics of QSM.

Previous studies have not reported the differences in quantitative susceptibility between the two diseases. To our knowledge, this is the first study that provides a comprehensive difference quantification of MS and NMOSD using QSM-derived radiomic features of the DGM. In our study, we extracted the most important radiomic features to establish the radiomics-only models. Furthermore, after fivefold cross-validation, we found that the DGM regions except for SN can differentiate the two diseases accurately with the AUC superior to 0.80. It indicated that the QSM-derived radiomic features do have the differences between MS and NMOSD patients and provide complementary information for clinical differential diagnosis of these two diseases.

In addition, the radiomics-only models based on the DN had the highest discrimination performance with an AUC of 0.902, using four radiomic features after feature selection. The gray-level run length matrix (GLRLM) gives the size of homogeneous runs in four directions for each gray level, and the gray-level zone length matrix (GLZLM) provides information on the size of homogeneous zones for each gray level in two dimensions. As the largest nucleus in the cerebellum, DN is located deep in the white matter of the cerebellum and has the functions of regulating body balance, regulating muscle tension, and coordinating voluntary movement. In MS patients, evidence has been found that the brain iron level is abnormal because iron accumulates in the basal ganglia area. Thus, we speculated that MS and NMOSD patients might have significant differences in athletic performance. Although the radiomic feature differences have been shown by DN in QSM images, there is no evidence that the two diseases have significant differences in the values of iron deposition in this area, which may be due to the long-term accumulation of iron deposition resulting in the changes in the images. However, due to the similarity of damage between the two diseases, the differences in quantitative susceptibility were not shown in previous studies.

The demographic information including patient age, sex, disease duration, and EDSS score was also collected in this work. The NMOSD patients showed a greater female predominance and higher EDSS scores than the MS patients. The demographic information-only model showed a lower AUC of 0.733 than any radiomics-only models in DGM regions except for SN. Combining the demographic information and QSM-derived radiomic features, we built a hybrid model that improved the discrimination performance and achieved the highest AUC of 0.927, highlighting the importance of comprehensive consideration of clinical and imaging features. Future work needs to clarify the underlying pathophysiological mechanism of the difference in QSM radiomic features between these two diseases, verify the effectiveness of this model, and promote the application of this model in clinical practice.

Several limitations also exist in this work. First, although we have extracted highly correlated radiomic features and obtained a relatively accurate discrimination performance, the pathophysiological mechanism is unclear. Second, our study only adopted QSM images reflecting iron deposition characteristics but did not include other sequences. Future work can use more sequences to obtain more information to identify MS and NMOSD. Finally, the relatively small sample size would affect the results of the discrimination model.

## Conclusion

In conclusion, we have investigated the performance of QSM-derived radiomic features of the DGM to distinguish MS from NMOSD. The results of our study indicate that the discrimination model with the QSM-derived radiomic features in combination with demographic information has the potential to classify MS and NMOSD.

## Data Availability Statement

The raw data supporting the conclusions of this article will be made available by the authors, without undue reservation.

## Ethics Statement

The studies involving human participants were reviewed and approved by the Institutional Review Board of the First Affiliated Hospital of Chongqing Medical University. The patients/participants provided their written informed consent to participate in this study.

## Author Contributions

ZY offered the researches idea and prepared the manuscript. HL offered the data processing support, which platforms and resources were assisted by the company. XC assisted in imaging processing. YZ assisted us in completing the statistics and experimental design. QZ, CZ, SD, YP, and YL provided guidance and critical reviews. All authors contributed to the article and approved the submitted version.

## Conflict of Interest

HL was employed by the company GE Healthcare, China. The remaining authors declare that the research was conducted in the absence of any commercial or financial relationships that could be construed as a potential conflict of interest.

## Publisher’s Note

All claims expressed in this article are solely those of the authors and do not necessarily represent those of their affiliated organizations, or those of the publisher, the editors and the reviewers. Any product that may be evaluated in this article, or claim that may be made by its manufacturer, is not guaranteed or endorsed by the publisher.

## Footnotes

1
https://people.eecs.berkeley.edu/~chunlei.liu/software.html

2
https://fsl.fmrib.ox.ac.uk/fsl/fslwiki/

## Abbreviations

MS: Multiple sclerosis
NMOSD: Neuromyelitis optica spectrum disorder
QSM: Quantitative Susceptibility Mapping
PUT: Putamen
GP: Globus pallidus
HCN: Head of the caudate nucleus
THA: Thalamus
SN: Substantia nigra
RN: Red nucleus
DN: Dentate nucleus
ROC: Receiver operating characteristic
AUC: Area under the receiver operating characteristic curve
MRI: Magnetic resonance imaging
EDSS: Expanded disability status scale
DGM: Deep gray matter.
